# Clinical epidemiology, risk factors and treatment outcomes of extended-spectrum beta-lactamase producing Enterobacteriaceae bacteremia among children in a Tertiary Care Hospital, Bangkok, Thailand

**DOI:** 10.1186/s13104-018-3729-3

**Published:** 2018-08-29

**Authors:** Thirapa Nivesvivat, Phunlerd Piyaraj, Sudaluck Thunyaharn, Veerachai Watanaveeradej, Detchvijitr Suwanpakdee

**Affiliations:** 1 Department of Pediatrics, Phramongkutklao Hospital, Bangkok, Thailand; 2 Department of Parasitology, Phramongkutklao College of Medicine, Bangkok, Thailand; 3 Faculty of Medical Technology, Nakhon Ratchasima College, Nakhon Ratchasima, Thailand; 4 Department of Microbiology, Phramongkutklao College of Medicine, Bangkok, Thailand

**Keywords:** ESBL-producing Enterobacteriaceae, Bacteremia, Paediatric

## Abstract

**Objective:**

Extended-spectrum beta-lactamase (ESBL) producing Enterobacteriaceae infection is an emerging problem in paediatric populations leading to increased mortality. The purpose of this study was to determine the prevalence, risk factors and clinical outcomes of ESBL-producing Enterobacteriaceae in paediatric blood stream infections (BSIs). A retrospective review of paediatric patients diagnosed with Enterobacteriaceae bacteremia was performed at Phramongkutklao Hospital from 2010 to 2017.

**Results:**

Among 97 non-duplicated blood isolates, the prevalence of ESBL-producing Enterobacteriaceae was 53.6% (28.9% *Escherichia coli* and 25.8% *Klebsiella* spp. isolates). The study indicated that the prevalence of ESBL infection was higher among patients with chronic illness, especially hematologic malignancies, than among patients without underlying disease (P = 0.01). No differences were observed in the prior use of any antibiotics, the use of extended-spectrum cephalosporin, neutropaenia or the presence of an indwelling central venous catheter. Mortality in the ESBL group was significantly higher than that in the non-ESBL group, with observed mortalities of 38.9% and 13.3%, respectively (P < 0.05). In conclusion, BSIs with ESBL-producing Enterobacteriaceae tended to increase infection rates and impact survival rates among paediatric patients.

## Introduction

Gram-negative blood stream infections (BSIs) caused by Enterobacteriaceae are a leading cause of severe and life-threatening conditions in paediatric bacteremia. *Escherichia coli* and *Klebsiella* spp. are the most common pathogens. Currently, broad-spectrum cephalosporins are widely administered for empirical treatment among children suffering from Gram-negative bacteremia. Therefore, multidrug-resistant strains of Enterobacteriaceae have emerged and have become an issue of concern worldwide. *Escherichia coli* and *Klebsiella* spp. are extended-spectrum beta-lactamase enzyme (ESBL) producing Enterobacteriaceae and multidrug-resistant Gram-negative organisms. These organisms are resistant to broad-spectrum cephalosporins, which affects treatment outcomes of BSIs among adults as well as children with bacteremia. ESBL-producing Enterobacteriaceae infections are increasingly being recognized among paediatric patients in various regions around the world, including America, Europe and Asia [1–4]. Several reports of outbreaks have been reported in newborn, paediatric and paediatric oncology wards [5]. Severe infections have been reported among newborns and patients with concomitant diseases such as leukaemia [1, 2]. However, limited clinical data are available concerning the epidemiology of ESBL bacteremia and its risk factors and treatment outcomes among children in developing countries in SE Asia. Therefore, the objective of this study was to determine the prevalence of ESBL-producing Enterobacteriaceae and its risk factors and treatment outcomes among paediatric patients suffering from ESBL bacteremia in a tertiary public care centre in Bangkok, Thailand.

## Main text

### Materials and methods

#### Study design and data analysis

A retrospective study was conducted to explore the total number of episodes of bacteremia caused by *Escherichia coli* and *Klebsiella* spp. among paediatric patients aged 0–18 years between January 2010 and September 2017 at Phramongkutklao Hospital, a tertiary care centre with 200 paediatric beds located in Bangkok, Thailand. Demographic characteristics were collected from medical records, including sex, age, clinical immune status, focal infections, underlying diseases, previous use of antibiotics, especially extended-spectrum cephalosporins, and final treatment outcomes. The study protocol was approved prior to study commencement by the Institutional Review Board of the Royal Thai Army Medical Department and a waiver of consent was granted.

Patient data and risk factors between the ESBL group and the non-ESBL group were compared using the Chi squared test, Fisher’s exact test or the Mann–Whitney U test as appropriate. The significance level was considered to be a P value of less than 0.05. The independent risk factors associated with ESBL were determined using multiple logistic regression analyses, and SPSS software, version 22.0 was used to perform the analyses.

#### Microbiological procedures

All *E. coli* and *Klebsiella* spp. bacteria were identified according to the Cowan and Steel method of bacteria identification [6]. Antibiotic susceptibility patterns were determined using the disk diffusion method according to the methods recommended by the National Committee for Clinical Laboratory Standards Institute (CLSI), and categorical assignments were made using CLSI breakpoints [7]. ESBL production was screened for and confirmed using a profile suggestive of resistance by performing a double-disc synergy test according to CLSI guidelines [7].

### Results

**Fig. 1:**
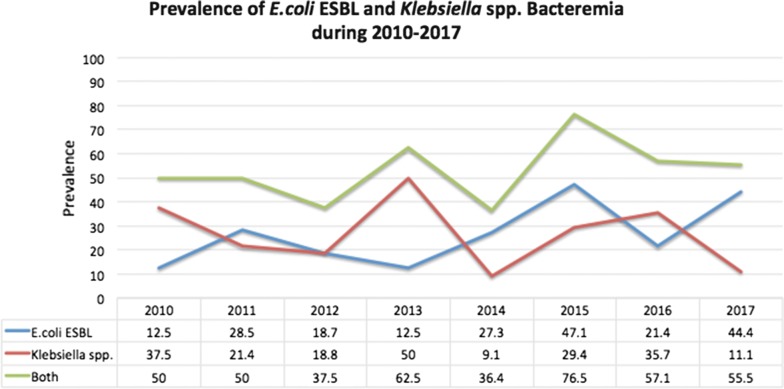
Prevalence of *E. coli* ESBL and *Klebsiella* spp. Bacteremia during 2010–2017

Out of all of the haemoculture specimens obtained from January 2010 to September 2017, 369 cases were caused by Gram-negative bacilli bacteremia, and 97 (26.2%) were caused by Enterobacteriaceae bacteria. The prevalence of ESBL-producing Gram-negative bacilli bacteremia was 52 out of 97 episodes (53.6%), including 28.9% of the *E. coli* and 25.8% of the *K. pneumoniae* specimens. The prevalences of ESBL *E. coli* and *Klebsiella* spp. are shown in Fig. 1. The prevalence of the ESBL-producing group was highest (76.5%) in 2015 and slightly decreased to 64.29 and 55.56% in the following 2 years, respectively. Rates of the use of any antibiotics and extended-spectrum cephalosporin specifically within the previous 30 days were also highest (64.6 and 47.5%, respectively) in 2015 and decreased in following years.

The demographic data and clinical characteristics for the two groups are shown in Table 1. The mean ages of the two groups of patients were 65 ± 81 and 69 ± 75 months, respectively. The length of stay for the ESBL group was 93 ± 180 days, which was longer than the length of stay for the non-ESBL group, which was 37 ± 57 days. Patients who had underlying diseases had a higher prevalence of ESBL bacteremia than those without underlying conditions (P < 0.01). Common underlying diseases in the ESBL group were hematologic malignancies (28.8%), congenital heart disease (23.0%) and neurological problems (7.6%), while most Enterobacteriaceae bacteremia among pre-term infants and newborns were found in the non-ESBL group (90%).

**Table 1:** Demographic and clinical feature at the time of presentation of children with bacteremia from ESBL group and non ESBL group *E. coli* or *Klebsiella* spp.

	Non-ESBL (n=45)	ESBL (n=52)	P-value
Sex (M:F)	26:19	27:25	0.334
Mean ± SD of age (month)	65 ± 81	69 ± 75	0.708
Mean ± SD of length of stay	37 ± 57	93 ± 180	0.083
Day	25	40	< 0.01
Underlying disease			
Hematological malignancy	8	15	0.463
Solid tumor	2	2	0.661
Nephrotic syndrome	2	3	1
Congenital heart disease	1	12	< 0.01
End stage renal disease	3	3	0.661
SLE	0	2	0.497
Preterm	7	0	< 0.01
Newborn	2	1	0.595
Chronic lung disease	0	2	0.497
Neurological problem	4	4	0.729
Focal infection			
Pneumonia	10	21	0.17
Urinary tract infection	13	14	0.58
Diarrhea	1	5	0.18
Cellulitis	1	3	0.54
Ascending cholangitis	2	0	0.15
Meningitis	2	2	0.72
Mortality (%)	6 (13.3%)	20 (38.9%)	< 0.05

ESBL group: patient with bacteremia caused by ESBL producing—*E. coli* or *Klebsiella* spp.

Non-ESBL group: patient with bacteremia caused by non-ESBL producing—*E. coli* or *Klebsiella* spp.

Eighty-seven percent (45 of 52) of patients in the ESBL-producing bacteremia group had focal infections including pneumonia (40.3%), urinary tract infections (26.9%), meningitis (3.8%), diarrhoea (9.6%) and cellulitis (5.7%). The prevalences of focal infections in the ESBL and non-ESBL groups were not significantly different. The mortality rates in the ESBL and non-ESBL groups were 38.9% and 13.3%, respectively (P < 0.05). In the ESBL group, 29 cases (55.8%) were treated using carbapenem or carbapenem with aminoglycoside, while only 5 cases (9%) were treated using aminoglycoside or quinolone. No differences in mortality rates were found between the two groups. Eight palliative patients did not receive any antibiotics, and ten of the ESBL cases had missing data.

The risk factors for BSIs caused by ESBL-producing strains of *E. coli* and *K. pneumoniae* were analysed using a univariate analysis, which found that they included the use of any antibiotics within 30 days, the use of extended-spectrum cephalosporin within 30 days, the presence of an indwelling central venous catheter, and neutropaenia (ANC < 500). A univariate analysis revealed that the risk factors significantly associated with ESBL-producing *E. coli* and *Klebsiella* spp. included the use of any antibiotics within 30 days (OR 5.4; 95% CI, 2.3–13) and the use of extended-spectrum cephalosporin within 30 days (OR 5.8; 95% CI, 2.2–15.5) (Table 2).

**Table 2:** Analysis of Risk factors for bacteremia caused by ESBL-producing group versus ESBL-nonproducing *E. coli* or *Klebsiella* spp.

	Non ESBL	ESBL	P-value	Crude odds ratio	95% CI	Adjusted odds ratio	95% CI
Use of any antibiotics within previous 1 mo	15 (33.3)	38 (73.1)	< 0.05	5.4	2.3–13.0	2.7	0.8–9.3
Use of extended-spectrum cephalosporin within previous 1 mo	7 (15.6)	27 (51.9)	< 0.05	5.8	2.2–15.5	2.4	0.7–9.0
Neutropenia (ANC < 500)	8 (17.8)	17 (34)	0.077	2.4	0.9–6.2	2.5	0.9–7.5
Presence of an indwelling central venous catheter	11 (34.4)	21 (40.4)	0.099	2.1	0.9–5.1	1.6	0.6–4.8

A logistic regression analysis showed no significant association between bacteremia caused by the ESBL-producing group and two variables: the use of any antibiotics within 30 days (OR 2.7; 95% CI, 0.8–9.3) and the use of extended-spectrum cephalosporin within 30 days (OR 2.4; 95% CI, 0.7–9.0) (Table 2).

### Discussion

Despite the fact that ESBL-producing Enterobacteriaceae bacteremia is emerging as an important worldwide multidrug resistance problem, we currently lack clinical epidemiology data from resource-limited countries. A recent study in northeast Thailand reported that *E. coli* and *K. pneumoniae* were the most common Gram-negative bacteremia. From 2004 to 2010, the proportions of ESBL-producing *E. coli* and *K. pneumoniae* were 30.4% and 33.2%, respectively, and an increase in the rate of ESBL-producing *E. coli* from 33.3 to 51.5% was reported, [8]. In comparison, we observed a high prevalence of ESBL-producing Gram-negative bacilli bacteremia (53.6%) among paediatric patients. These findings indicated that ESBL-producing Enterobacteriaceae bacteremia have an actual negative impact among adult and paediatric populations living in resource-limited countries such as Thailand.

In our study, the highest prevalence of ESBL bacteremia was reported in 2015, which can be explained by the high rate of previous antibiotics exposure that was reported in the same year. After this report, we implemented a strict antimicrobial stewardship program to reduce excessive antibiotics prescriptions among paediatric inpatients. We also enhanced contact precautions, especially for hand hygiene, and provided education, including demonstrations of infection and control protocols, for healthcare providers. This was shown to be a valuable infection control policy, as the prevalence of ESBL bacteremia decreased in the following year (Fig. 1). However, it seems that *E. coli* bacteremia increased again due to natural variation in other years.

A history of antibiotic use, especially the use of third-generation cephalosporins, a history of *Klebsiella* spp. infection and being female were reported in related studies as factors commonly associated with ESBL infection [3, 9]. Similarly, using a univariate analysis, our results revealed that the use of any antibiotics within 30 days and the use of extended-spectrum cephalosporin within 30 days were factors associated with ESBL group infection. However, both risk factors were not significantly associated, as was proven through a logistic regression analysis (OR 1.8, 2.6 and 95% CI 0.5–6.1 and 0.7–9.6, respectively). These findings were affected by the limitations of the sample size. Kuo et al. [3] and Somily et al. [10] reported that the insertion of a central venous line was a significant risk factor associated with ESBL infection, which is in contrast to our results.

Kim et al. [11] found that use of vancomycin antibiotics for infants in an intensive care unit was associated with ESBL group infection, but this association was not found in our report.

Similarly, regarding focal sites of infections associated with ESBL-producing BSIs, pneumonia (40%) and urinary tract infections (27%) were the leading causes of coexistent infections, which is similar to the results of a related study that reported pneumonia (46%) and urinary tract infections (23%) as leading causes [11]. These were two common focal infections for both ESBL and non-ESBL bacteremia.

While carbapenem is the antimicrobial of choice among children suffering from ESBL-producing Enterobacteriaceae bacteremia, we found that some isolates were susceptible to quinolone or aminoglycoside, and favourable response rates were shown in our series. Even though most patients received a timely diagnosis and were treated with carbapenem, the overall mortality rate in the ESBL group was significantly higher than that in the non-ESBL group; this was similar to findings in a related report among children [11, 12]. The clinical responses and mortality rates did not differ between patients receiving carbapenem as monotherapy and patients receiving carbapenem plus aminoglycoside. Therefore, a combined regimen has not been recommended for patients infected with ESBL-producing Enterobacteriaceae.

In conclusion, this study found a high prevalence of ESBL-producing Enterobacteriaceae among paediatric BSIs at our institute in Thailand as well as worldwide. These findings require special attention due to the high morbidity and mortality rates observed. The appropriate antibiotic, carbapenem, should be started in patients suspected to have a BSI from an ESBL-producing organism, and infection control policies, such as antibiotic stewardship programs and transmission-based precautions, should be promoted to prevent the spread of these multidrug resistant infections.

## Limitations

This study was conducted in a single centre at a Tertiary Care Hospital in Bangkok, Thailand. Therefore, the high prevalence found in our results does not represent the actual prevalence of ESBL-producing bacteremia in the paediatric population in Bangkok. We propose that multicenter surveillance be conducted in the future to estimate the existing impact of these problems in our region. In addition, our small sample size may have impacted non-significant findings in our study, and the data among infants were limited. Thus, a surveillance study in the newborn population is recommended in the future.
